# Identification of characteristics predictive of long-term survival with durvalumab or durvalumab plus tremelimumab in metastatic urothelial carcinoma

**DOI:** 10.1186/s12885-023-11380-6

**Published:** 2023-09-29

**Authors:** Marie Alt, Carlos Stecca, Yian Lin, Gbenga Kazeem, Erik T. Goluboff, Srikala S. Sridhar

**Affiliations:** 1 Princess Margaret Cancer Centre, Princess Margaret Hospital, University of Toronto, Toronto, ON Canada; 2 Present Address: Centre Hospitalier de Haguenau, Haguenau, France; 3 AstraZeneca, San Francisco, CA USA; 4 AstraZeneca, Cambridge, UK; 5 AstraZeneca, Gaithersburg, MD USA

**Keywords:** Durvalumab, Immune checkpoint inhibitor, Long-term survival, Multivariable analysis, Predictive factors, Univariable analysis, Urothelial carcinoma

## Abstract

**Background:**

This retrospective analysis of data from clinical trials in metastatic urothelial carcinoma (mUC) was conducted to determine baseline patient characteristics associated with long-term survival (LTS) following treatment with immune checkpoint inhibitors.

**Methods:**

Data for this analysis were from patients with platinum-refractory mUC who received durvalumab or durvalumab plus tremelimumab in phase 1/2 studies. The primary outcome measure was LTS. Patients were categorised as overall survival (OS) ≥ 2 years (from first dose) or OS < 2 years. A univariable analysis assessed independent associations with LTS and multivariable logistic regression was employed including each variable with *P* ≤ 0.05 as covariates.

**Results:**

Among 360 patients, 88 (24.4%) had OS ≥ 2 years and 272 (75.6%) had OS < 2 years. In univariable analysis, several baseline characteristics and laboratory measurements were associated with LTS including sex, ECOG PS, PD-L1 expression, prior surgery, time from initial diagnosis, lymph node-only involvement, visceral disease, haemoglobin level, absolute neutrophil count, neutrophil–lymphocyte ratio and lactate dehydrogenase level. In multivariable analysis, LTS was significantly associated with ECOG PS, PD-L1 expression, haemoglobin level and absolute neutrophil count.

**Conclusions:**

Several baseline clinical characteristics and laboratory measurements were associated with LTS for patients with platinum-refractory mUC treated with durvalumab or durvalumab plus tremelimumab.

**Supplementary Information:**

The online version contains supplementary material available at 10.1186/s12885-023-11380-6.

## Background

Platinum-based chemotherapy remains the standard of care for the first-line treatment of metastatic urothelial carcinoma (mUC), but outcomes are poor with a median overall survival (OS) ranging from 12 to 15 months [1–3]. While objective response rates (ORRs) are high with platinum-based chemotherapy (44%–49%), median duration of response is typically only 6–8 months [1–3]. Use of single-agent anti-programmed cell death-1 (PD-1) or anti-programmed cell death ligand-1 (PD-L1) agents in previously untreated patients with mUC yields ORRs of 23%–29% and median OS of 11.3 to 15.9 months [1–6]. In platinum-refractory mUC, ORRs of 15%–21% and median OS of 7.9 to 11.1 months have been reported with anti–PD-1/PD-L1 agents [7–12]. Compared with chemotherapy, longer durations of response are consistently observed with anti–PD-1/PD-L1 agents, with a higher proportion of patients experiencing long-term survival outcomes [1–3, 7, 9]. In updated results from the KEYNOTE-045 study in platinum-refractory mUC, median duration of response was 29.7 months with pembrolizumab and 4.4 months with chemotherapy and 3-year OS rates were 20.7% and 11.0%, respectively [13]. Despite robust responses in a proportion of patients, outcomes with the immune checkpoint inhibitors (ICIs) are highly variable and factors that can predict durable clinical benefit are yet to be defined.

There is evidence in mUC to suggest that high tumour PD-L1 expression may enrich for response to anti–PD-1/PD-L1 agents, leading to a prolonged OS [1–3, 8, 10]. However, inconsistent results have been reported, particularly in the second-line setting where high tumour PD-L1 expression has been shown to enrich for response to nivolumab [12] and durvalumab [8, 10] but not to pembrolizumab [7] or atezolizumab [9]. While the role of PD-L1 in mUC continues to be evaluated, several clinical characteristics have been identified as potential prognostic factors for OS. The original risk score proposed by Bajorin et al. [14] for patients treated with cisplatin-based chemotherapy, which included performance status (PS) and visceral metastasis as independent prognostic factors for OS, was later expanded to a four-variable model that included PS, visceral metastasis, albumin and haemoglobin [15]. In platinum-refractory mUC, Bellmunt et al. [16] identified Eastern Cooperative Oncology Group (ECOG) PS ≥ 1, haemoglobin level < 10 g/dL and presence of liver metastases as the main adverse prognostic factors for OS. In the first-line setting, Khaki et al. [17] recently proposed a prognostic model in which ECOG PS ≥ 2, albumin < 3.5 g/dL, neutrophil–lymphocyte ratio (NLR) > 5 and liver metastases were used to derive a risk score for OS. Using patient-level data from clinical studies of atezolizumab, avelumab and durvalumab in platinum-refractory mUC, Sonpavde et al. [18] developed a prognostic model for OS that includes ECOG PS (1 vs 0), liver metastasis, platelet count, NLR and lactate dehydrogenase (LDH) levels.

The current retrospective analysis was undertaken to identify factors that may predict long-term benefit with ICIs in mUC. We evaluated several baseline characteristics among long-term and short-term survivors who received durvalumab (PD-L1 inhibitor) monotherapy or durvalumab combined with tremelimumab (anti–cytotoxic T-lymphocyte antigen 4 inhibitor) in the platinum-refractory setting.

## Methods

### Analysis population

The primary analysis population included 192 patients from Study 1108 and 168 patients from Study 10 who were enrolled in the studies at the time of this analysis. Additional patients continued to be enrolled in Study 1108, however, these patients were not included in the current analysis. Study 1108 (NCT01693562) was a phase 1/2, multicentre, open-label study of durvalumab monotherapy at a dose of 10 mg/kg every 2 weeks for up to 12 months in solid tumors. Eligible patients could have disease that progressed on prior therapy or be treatment-naïve, had ECOG PS 0/1, and had adequate organ and bone marrow function. Patients were not eligible if they had received any immunotherapy or investigational anti-cancer therapy within the past 4 weeks (6 weeks for monoclonal antibodies) or if they were receiving any concurrent chemotherapy, immunotherapy, biologic, or hormonal therapy for cancer. Patients who met these criteria and had locally advanced/metastatic UC and whose disease had progressed while they were receiving prior therapy or were ineligible for or refused any number of prior therapies were eligible for inclusion in the UC cohort. Patients from the UC cohort were included in the current analysis [8, 10].

Study 10 (NCT02261220), was a phase 1, multicentre, open-label study of durvalumab at 20 mg/kg plus tremelimumab at 1 mg/kg every 4 weeks for 4 months, followed by durvalumab monotherapy at 10 mg/kg every 2 weeks for up to 12 months in solid tumours [19]. Eligible patients had histologic confirmation of advanced solid tumours, recurrent/metastatic disease and may have previously been treated in the recurrent/metastatic setting. Patients were excluded if they had used any concurrent chemotherapy, immune-mediated therapy or biologic or hormonal therapy for cancer treatment, had active or prior documented autoimmune disease within the past 2 years, or if there was current or prior use of immunosuppressive medication within 14 days. Patients from this UC cohort were included in the current analysis.

This is a post hoc analysis of data previously collected from Study 1108 and Study 10. The data involved included demographic, survival data and tumour response. Data were already available for analysis, with no new data collected from the patients and thus the original study consent forms covered this analysis. The inclusion of patients’ data in this post hoc analysis was allowed under the Study 1108 and Study 10 consent forms. Study 1108 and Study 10 were conducted according to the Declaration of Helsinki and approved by the independent ethics committee or institutional review board at each of the participating centres (Supplementary Table 1), with written informed consent obtained from all patients. All methods were carried out in accordance with relevant guidelines and regulations. The analysis population included all patients who received at least one dose of durvalumab, had received prior platinum therapy as first-line chemotherapy, had a baseline assessment with measurable disease (per blinded independent central review according to Response Evaluation Criteria in Solid Tumors, version 1.1 [RECIST v1.1]), and at least 24 weeks of follow-up at the time of data cutoff on September 22, 2020.

### Data collection and outcome measures

Demographic, clinicopathologic, radiologic and laboratory data at baseline were collected for all patients. The primary outcome measure was long-term survival, defined as OS of ≥ 2 years from the date of first dosing with durvalumab or durvalumab plus tremelimumab. OS was defined as the time from start of treatment to date of death from any cause. For patients who were alive at the time of data cutoff or lost to follow-up, OS was censored on the date when the patients were known to be alive. For the purpose of these analyses, patients were categorised as OS ≥ 2 years or OS < 2 years without consideration of censoring status. A sensitivity analysis was conducted where censored patients were excluded from the OS < 2 years subgroup. Long-term progression-free survival (PFS), defined as a PFS duration of ≥ 6 months from the start of treatment until objective progression or death, was a secondary outcome measure. Other secondary outcome measures were investigator-assessed objective response according to RECIST v1.1 and duration of response. Baseline PD-L1 expression was assessed in formalin-fixed, paraffin-embedded tumour samples by immunohistochemistry using the VENTANA PD-L1 (SP263) Assay (Ventana Medical Systems, Tucson, AZ, USA) [20]. PD-L1 expression was defined as “high” if ≥ 25% of tumour cells or ≥ 25% of tumour-infiltrating immune cells exhibited membrane staining [10].

### Univariable and multivariable statistical analyses

A univariable analysis was conducted on each baseline characteristic in order to assess its independent association with long-term survival. Continuous variables are summarised by descriptive statistics, which included number of patients, mean and standard deviation. Categorical data are summarised by the number and percentage of patients in each category. For continuous variables, the *P* value was obtained from the t-test statistic. For categorical variables, the chi-square test statistic was used to perform the assessment; Fisher’s exact test was used for categorical variables where the number of patients was < 5 in any comparison group. The percentage of patients achieving an OS of ≥ 2 years at the time of data cutoff were analysed using a multivariable logistic regression analysis including variables identified in the univariable analysis with *P* ≤ 0.05 as covariates. For each independent variable (predictor) included in the analysis, the odds ratio, two-sided 95% confidence interval (CI) and *P* value were calculated. Similarly, the percentage of patients achieving PFS of ≥ 6 months at the time of data cutoff were analysed using a multivariable logistic regression analysis. All analyses were post hoc and exploratory, and thus no adjustment for multiple testing was performed. Median OS and median PFS were calculated using the Kaplan–Meier method. The difference in ORRs between the OS ≥ 2 years and OS < 2 years groups was compared using the chi-square test. All statistical analyses were conducted using SAS v 9.4 software (SAS Institute, Cary, NC, USA).

## Results

### Primary analysis population and OS

**Fig. 1:**
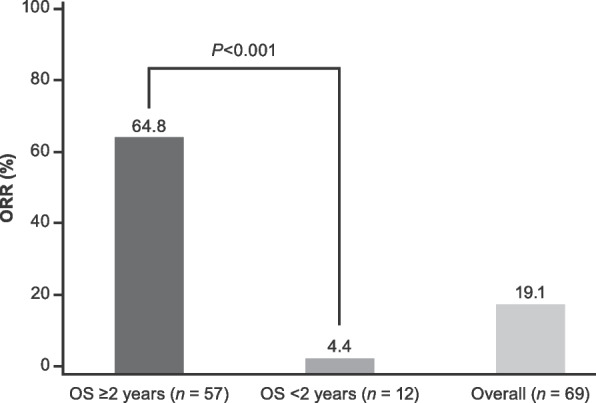
ORR by OS subgroups. Tumour response was assessed in all evaluable patients (88 in the OS ≥ 2 years subgroup and 212 in the OS < 2 years subgroup). For each OS subgroup, the total number of patients who achieved a complete or partial response is given in parentheses. *ORR* objective response rate, *OS* overall survival

Among the 360 patients with mUC included in the analyses, 88 (24.4%) had an OS ≥ 2 years and 272 (75.6%) had an OS < 2 years. Most patients (355; 98.6%) had at least one prior line of therapy, 275 patients (76.4%) had prior surgery, 84 patients (23.3%) had prior radiotherapy, 59 patients (16.4%) had used a biologic and 10 patients (2.8%) had used a prior immunotherapy (Table 1). Sixty-two of the 88 patients (70.5%) in the OS ≥ 2 years subgroup completed treatment whereas only 7 of 272 patients (2.6%) in the OS < 2 years subgroup completed treatment (Supplementary Table 2). The primary reason for discontinuation of treatment was disease progression (17.0% in the OS ≥ 2 years subgroup and 68.4% in the OS < 2 years subgroup). By the Kaplan–Meier method, median OS was not reached (NR; 95% CI, 57.4–NR) in the OS ≥ 2 years subgroup, was 5.4 months (95% CI, 4.2–6.9) in the OS < 2 years subgroup, and was 9.6 months (95% CI, 7.9–12.5) in the total population. Patients with an OS ≥ 2 years had a significantly higher ORR than those with an OS < 2 years (Fig. 1) and a significantly longer median duration of response at15.1 months versus 1.5 months, respectively (*P* < 0.001).

**Table 1:** Univariable analysis of associations between baseline patient demographic/clinicopathologic characteristics and long-term survival

Variable	OS ≥ 2 years (*n* = 88)	OS < 2 years (*n* = 272)	*P* value^a^
Sex –* n* (%)			0.020
Female	14 (15.9)	77 (28.3)	
Male	74 (84.1)	195 (71.7)	
Age, years – mean (SD)	64.8 (8.3)	65.8 (9.9)	0.353
Age group – *n* (%)			0.518
< 50 y	3 (3.4)	17 (6.3)	
≥ 50– < 60 y	21 (23.9)	48 (17.6)	
≥ 60– < 70 y	35 (39.8)	109 (40.1)	
≥ 70 y	29 (33.0)	98 (36.0)	
Race – *n* (%)			0.191
Asian	11 (12.5)	56 (20.6)	
Black or African American	1 (1.1)	9 (3.3)	
White	66 (75.0)	173 (63.6)	
Other	5 (5.7)	20 (7.4)	
Unknown	5 (5.7)	14 (5.1)	
Smoking history – *n* (%)			0.786
Never	34 (38.6)	95 (34.9)	
Former	45 (51.1)	150 (55.1)	
Current	9 (10.2)	26 (9.6)	
Unknown	0	1 (0.4)	
Pack years smoked – mean (SD)^b^	20.7 (28.1)	24.5 (32.7)	0.362^c^
ECOG PS – *n* (%)			< 0.001
0	50 (56.8)	77 (28.3)	
1 or 2	38 (43.2)	195 (71.7)	
Prior lines of treatment – *n* (%)			0.075
0	3 (3.4)	2 (0.7)	
1	58 (65.9)	170 (62.5)	
2	22 (25.0)	87 (32.0)	
3	5 (5.7)	7 (2.6)	
4	0 (0.0)	6 (2.2)	
Prior surgery – *n* (%)			0.005
Yes	77 (87.5)	198 (72.8)	
No	11 (12.5)	74 (27.2)	
Prior radiotherapy – *n* (%)			0.189
Yes	16 (18.2)	68 (25.0)	
No	72 (81.8)	204 (75.0)	
Prior biologic – *n* (%)			0.065
Yes	20 (22.7)	39 (14.3)	
No	68 (77.3)	233 (85.7)	
Prior immunotherapy – *n* (%)			1.000
Yes	2 (2.3)	8 (2.9)	
No	86 (97.7)	264 (97.1)	
Prior cisplatin-based regimen – *n* (%)			0.915
Yes	66 (75.0)	196 (72.1)	
No	15 (17.0)	43 (15.8)	
Missing	7 (8.0)	33 (12.1)	
Prior carboplatin-based regimen – *n* (%)			0.917
Yes	25 (28.4)	82 (30.1)	
No	31 (35.2)	105 (38.6)	
Missing	32 (36.4)	85 (31.3)	
PD-L1 status – *n* (%)^d^			0.001
Low or negative	26 (29.5)	140 (51.5)	
High	52 (59.1)	115 (42.3)	
Missing	10 (11.4)	17 (6.3)	
Time from initial diagnosis to study entry, months – mean (SD)	34.3 (39.7)	27.4 (27.9)	0.047^c^
Lymph node-only involvement – *n* (%)			< 0.001
Yes	26 (29.5)	18 (6.6)	
No	62 (70.5)	254 (93.4)	
Visceral disease – *n* (%)			< 0.001
Yes	43 (48.9)	218 (80.1)	
No	45 (51.1)	54 (19.9)	

*ECOG PS* Eastern Cooperative Oncology Group performance status, *PD-L1* programmed cell death ligand-1, *PFS* progression-free survival, *SD* standard deviation

^a^*P* values were estimated from t-test for continuous variables, chi-squared for categorical variables where the number of patients was ≥ 5 in each comparison group, and Fisher’s exact test for categorical variables where the number of patients was ≤ 5 in any comparison group. Comparisons were based on non-missing data

^b^Number of packs per day × no. of years smoked.* n* = 72 for the OS ≥ 2 years subgroup and *n* = 199 for the OS < 2 years subgroup

^c^*P*-value was obtained from t-test based on log-transformed data

^d^PD-L1 expression was assessed by immunohistochemistry using the VENTANA PD-L1 (SP263) Assay. PD-L1 expression was defined as “high” if ≥ 25% of tumour cells or ≥ 25% of tumour-infiltrating immune cells had PD-L1 membrane staining. PD-L1 expression was defined as “low or negative” if < 25% of both tumour cells and immune cells had membrane staining for PD-L1

### Patient characteristics associated with long-term survival

Demographic and clinicopathologic characteristics of the patients at baseline are listed in Table 1. Patients in the OS ≥ 2 years subgroup had, overall, a better ECOG PS than patients in the OS < 2 years subgroup (56.8% vs 28.3% had an ECOG PS of 0 [*P* < 0.001], respectively). More patients in the OS ≥ 2 years subgroup had high tumour PD-L1 expression compared with the OS < 2 years subgroup (59.1% vs 42.3% [*P* 0.001]). In the OS ≥ 2 years subgroup, more patients had lymph node-only involvement compared with the OS < 2 years subgroup (29.5% vs 6.6% [*P* < 0.001]) and fewer patients had visceral disease (48.9% vs 80.1% [*P* < 0.001]). The results of the univariable analysis showed significant independent associations (*P* < 0.05) between long-term survival and sex, ECOG PS, prior surgery, PD-L1 expression, time from initial diagnosis to study entry, lymph node-only involvement and visceral disease (Table 1).

Baseline laboratory measurements evaluated in the primary analysis population are listed in Table 2. Absolute lymphocyte count, absolute eosinophil count and creatinine clearance were similar between the two OS subgroups. However, patients in the OS ≥ 2 years subgroup had higher baseline haemoglobin levels compared with patients in the OS < 2 years subgroup. In contrast, patients in the OS ≥ 2 years subgroup had lower baseline absolute neutrophil count, absolute monocyte count, ratio of NLR, ratio of neutrophils to leukocytes and LDH levels compared with patients in the OS < 2 years subgroup. By univariable analysis, significant independent associations (*P* < 0.05) with long-term survival were observed with haemoglobin level, absolute neutrophil count, absolute monocyte count, NLR, ratio of neutrophils to leukocytes and LDH level (Table 2).

**Table 2:** Univariable analysis of associations between baseline laboratory measurements and long-term survival

Variable^a^	OS ≥ 2 years (*n* = 88)	OS < 2 years (*n* = 272)	*P* value
Haemoglobin level (g/dL)	12.4 (1.7)	11.1 (1.4)	< 0.001
Absolute neutrophil count (10^3^/µL)	4.5 (2.5)	6.5 (4.4)	< 0.001^b^
Absolute monocyte count (10^3^/µL)	0.6 (0.3)	0.8 (0.4)	0.001^b^
Absolute lymphocyte count (10^3^/µL)	1.3 (0.6)	1.3 (0.6)	0.590^b^
Absolute eosinophil count (10^3^/µL)	0.2 (0.2)	0.2 (0.4)	0.267^b^
Neutrophil–lymphocyte ratio	4.2 (3.8)	6.6 (7.0)	< 0.001^b^
Neutrophils/leukocytes	66.4 (9.5)	71.0 (10.6)	0.001^b^
Creatinine clearance (mL/min)	71.9 (25.8)	68.0 (24.5)	0.199
Lactate dehydrogenase level (U/L)	253.2 (119.6)	370.4 (521.3)	0.003^b^

*OS* overall survival

^a^Values for laboratory measurements are mean (standard deviation)

^b^*P* value was obtained from t-test based on log-transformed data

Based on factors identified in the univariable analysis that had a *P* value ≤ 0.05, the results of the multivariable logistic regression analysis showed significant associations between long-term survival and several baseline characteristics and laboratory measurements – ECOG PS, PD-L1 expression, haemoglobin level and absolute neutrophil count (Table 3). A sensitivity analysis was conducted where censored patients were excluded from the OS < 2 years subgroup, and no substantial differences were observed (data not shown).

**Table 3:** Multivariable logistic regression model: associations between baseline characteristics and long-term survival

**Variable**^**a**^	**Odds ratio (95% CI)**	***P* value**
Sex		
Male	(Reference)	
Female	0.55 (0.25–1.23)	0.146
ECOG PS		
0	(Reference)	
1 or 2	0.43 (0.22–0.83)	0.012
Prior surgery		
No	(Reference)	
Yes	2.39 (0.97–5.89)	0.057
PD-L1 expression		
Low or negative	(Reference)	
High	2.23 (1.16–4.30)	0.017
Time from initial diagnosis to study entry^a^	1.06 (0.70–1.60)	0.789
Lymph node-only involvement		
No	(Reference)	
Yes	1.23 (0.40–3.79)	0.717
Visceral disease		
No	(Reference)	
Yes	0.55 (0.23–1.33)	0.182
Haemoglobin level	1.57 (1.22–2.01)	< 0.001
Absolute neutrophil count^a^	0.15 (0.03–0.69)	0.015
Absolute monocyte count^a^	1.48 (0.49–4.50)	0.488
Neutrophil–lymphocyte ratio^a^	1.70 (0.38–7.55)	0.483
Neutrophils/leukocytes^a^	4.42 (0.01–2345)	0.642
Lactate dehydrogenase level^a^	0.75 (0.39–1.47)	0.405

*ECOG PS* Eastern Cooperative Oncology Group performance status, *PD-L1* programmed cell death ligand-1

^a^Data were log-transformed for the logistic regression analysis

### Patient characteristics associated with long-term PFS

Among the 360 patients included in the analyses, 84 (23.3%) had a PFS ≥ 6 months and 276 (76.7%) had a PFS < 6 months. By the Kaplan–Meier method, median PFS was 43.6 months (95% CI, 19.9–NR) in the PFS ≥ 6 months group, 1.7 months (95% CI, 1.5–1.8) in the PFS < 6 months group, and 1.9 months (95% CI, 1.8–2.6) in the total population. The results of the univariable analysis showed significant independent associations (*P* < 0.05) between long-term PFS and ECOG PS, prior lines of treatment, prior surgery, prior biologic therapy, PD-L1 expression, lymph node-only involvement and visceral disease (Supplementary Table 3). For baseline laboratory measurements, significant independent associations with long-term PFS were observed with haemoglobin level, absolute neutrophil count, absolute monocyte count, NLR, ratio of neutrophils to leukocytes and LDH level (Supplementary Table 4). The results of the multivariable logistic regression analysis showed significant associations with long-term PFS and PD-L1 expression and absolute neutrophil count (Supplementary Table 5).

## Discussion

Among patients with mUC who had progressed on or after platinum-based chemotherapy and had received durvalumab or durvalumab plus tremelimumab, 24% survived 2 years or longer, with some patients surviving up to 5 years at the time of data cutoff. The results of our multivariable analysis showed that ECOG PS, PD-L1 expression, haemoglobin level and absolute neutrophil count were significantly associated with OS ≥ 2 years. Sex, prior surgery, time from initial diagnosis to study entry, lymph node-only involvement, visceral disease, absolute monocyte count, NLR, ratio of neutrophils to lymphocytes and LDH level showed significant independent associations with long-term survival in the univariable analysis but were not significantly associated with long-term survival when included in the multivariable logistic regression model. Although race was not associated with survival in the univariable analyses, it should be noted that the Black or African American population was underrepresented in this analysis. The incidence of mUC is approximately two times higher in the White population than in African Americans (23.1 vs 12.6 cases/100,000 persons) [21], however, in Study 1108 [10] 71% were White and only 4.6% Black or African American. Long-term survival was also significantly associated with ORR, which was markedly higher in patients who survived at least 2 years, and these patients had a much longer duration of response.

The proportion of patients with long-term survival and long-term PFS were similar, suggesting that patients whose disease does not progress within 6 months of treatment with ICIs can derive long-term durable benefit. In contrast to the results obtained for OS subgroups, sex and time from initial diagnosis to study entry were not significantly associated with long-term PFS in the univariable analysis, whereas prior biologic therapy and number of prior lines of treatment appeared to impact long-term PFS but not long-term survival. All baseline laboratory measurements that were significantly associated with long-term survival in univariable analyses were also significantly associated with long-term PFS. However, only PD-L1 expression and absolute neutrophil count remained significantly associated with long-term PFS in the multivariable analysis. The reasons for the observed differences between long-term survival and long-term PFS are not readily apparent and thus require further exploration.

The results of our multivariable analysis confirm published findings that an ECOG PS ≥ 1 is prognostic of poor survival outcomes in patients with platinum-treated mUC [16, 18]. In the first-line setting, the risk score proposed by Bajorin et al. [14] in 1999 and the expanded model in 2013 [15], both included PS as an independent prognostic factor for OS. The new risk score proposed by Khaki et al. [17] includes an ECOG PS of ≥ 2 as a significant prognostic factor for OS based on a multivariate analysis. Along with PS, visceral metastasis was included as an independent prognostic factor for OS in the risk score originally proposed by Bajorin et al. [14] and was one of the four variables included in the updated model (lung, liver, bone or other non-lymph node metastasis) [15]. The newer prognostic models for mUC proposed by Khaki et al. [17] in the first-line setting and Sonpavde et al. [18] in the second-line setting both include liver metastasis as an independent prognostic factor for OS. The results of our univariable analyses suggest that visceral disease is a prognostic factor for long-term survival and long-term PFS, although it was not significantly associated with either endpoint in multivariable analyses.

In contrast to the recently proposed prognostic models [17, 18], but consistent with the adverse prognostic factors for OS identified by Apolo et al. [15] and by Bellmunt et al. [16], we found that haemoglobin level was significantly associated with long-term survival in the multivariable analysis. We did not evaluate serum albumin levels in our analyses. However, while albumin was included in the prognostic models of Apolo et al. [15] and Khaki et al. [17], it was not included in the model by Sonpavde et al. [18], suggesting that it may be an important prognostic factor for OS in the first-line setting but not for platinum-treated mUC. In patients with advanced melanoma treated with ipilimumab (anti–CTLA-4 agent), elevated LDH levels have been shown to be predictive of poor long-term survival outcomes [22]. Subsequently, a prognostic score for OS was proposed in ipilimumab-treated patients with advanced melanoma, which included LDH, ECOG PS and number of organs involved [23]. LDH level may be an important prognostic factor for survival in bladder cancer [24] but it is typically not reported in clinical studies of ICIs in advanced disease. Based on the results of our univariable analysis and the model of Sonpavde et al. [18], baseline LDH levels may also have prognostic value for survival outcomes in platinum-treated mUC.

Baseline assessments of different immune cell types and changes during treatment with ICIs have been studied extensively for their prognostic and predictive roles, respectively, in several tumour types. It is now well recognised that NLR is an indicator of systemic inflammation that, when elevated, is associated with worse survival outcomes in various solid tumours [25, 26]. NLR is included in both of the recently proposed prognostic models for first- and second-line mUC [17, 18], with a ratio > 5 associated with worse OS outcomes. Consistent with these studies, we also observed that NLR was independently associated with long-term survival and PFS in univariable analyses, with patients in the OS ≥ 2 years and PFS ≥ 6 months subgroups having a mean ratio < 5 and those in the OS < 2 years and PFS < 6 months subgroups having a mean ratio > 5. In non-small cell lung cancer, a linked OS dropout model was used to determine significant factors for OS [27]. Among all factors tested, NLR was the most influential factor on the predicted 1-year survival rates (approximately 60% vs 30% with NLR below and above the median [4.56]). PD-L1 expression (tumour cells/immune cells ≥ 25%), LDH and durvalumab clearance (*P* < 0.01) were also significantly associated with OS.

The results of our multivariable analyses suggest that high tumour PD-L1 expression at baseline may be prognostic of both long-term PFS and long-term survival in platinum-refractory mUC. Recently updated results from the KEYNOTE-52 trial, with up to 5 years of follow-up, showed a median OS of 11.3 months and 4-year OS rate of 19.0% among all cisplatin-ineligible patients treated with pembrolizumab; however, in patients with high PD-L1 expression (defined as a combined positive score ≥ 10 [assessed as the number of PD-L1–stained tumour cells and immune cells relative to the total number of tumour cells]), median OS was 18.5 months with a 4-year OS rate of 31.9% [28]. Thus, PD-L1 expression at baseline may be an important consideration for future prognostic models of OS (as well as PFS) in mUC. Other biomarkers have yet to be evaluated in prognostic models of mUC. These include a four-gene IFN-γ-positive signature, which has been shown to be associated with improved survival in durvalumab-treated patients with mUC [29], with higher expression in PD-L1–positive tumours [30], and high tumour mutational burden, which is associated with better survival outcomes in patients with mUC treated with durvalumab plus tremelimumab [31].

Limitations of this analysis include its retrospective nature which may include some selection bias and unaccounted for confounding factors. Inclusion of a larger cohort and analysis of other potential predictors of long-term survival such as molecular biomarkers and response to prior treatment would provide further validity of the data. A comparison of the outcomes of durvalumab alone versus durvalumab plus tremelimumab, could be further evaluated to assess the added benefit of combination therapy.

## Conclusions

The results of our retrospective analyses have identified several baseline factors that are significantly associated with long-term survival outcomes in patients with platinum-treated mUC who received durvalumab or durvalumab plus tremelimumab in clinical studies. While a similar proportion of patients experienced long-term survival and long-term PFS in our analyses, differences in the characteristics associated with PFS and OS outcomes need to be further explored. Additional studies are required to develop a validated, unified prognostic model for mUC, and to determine whether different prognostic models are needed in the first- and second-line settings. Development of such prognostic models, potentially including biomarkers, could be used to select patients who will derive the most benefit from ICIs and could be used as stratification factors in future randomised clinical trials.

### Supplementary Information

**Additional file 1:** **Online supplementary table 1.** Institutional Review Board (IRB) and Institutional Ethics Committees (IEC). **Online supplementary table 2.** Patient disposition and treatment. **Online supplementary table 3.** Univariable analysis of associations between baseline patient demographic/clinicopathologic characteristics and long-term PFS. **Online supplementary table 4.** Univariable analysis of associations between baseline laboratory measurements and long-term PFS. **Online supplementary table 5.** Multivariable logistic regression model: associations between baseline characteristics and long-term PFS.

## Data Availability

The datasets generated and/or analysed during the current study are available via the Vivli platform. Data underlying the findings described in this manuscript may be obtained in accordance with AstraZeneca’s data sharing policy described at https://astrazenecagrouptrials.pharmacm.com/ST/Submission/Disclosure. Data for studies directly listed on Vivli can be requested through Vivli at www.vivli.org. Data for studies not listed on Vivli could be requested through Vivli at https://vivli.org/members/enquiries-about-studies-not-listed-on-the-vivli-platform/. The request will undergo an internal review process, and if approved, data will be prepared and shared with specified accessors named on the request form for 12 months via Vivli Secure Research Environment. AstraZeneca Vivli member page is also available outlining further details: https://vivli.org/ourmember/astrazeneca/.
