# Supplementary material for: Identification of characteristics predictive of long-term survival with durvalumab or durvalumab plus tremelimumab in metastatic urothelial carcinoma
Source: BMC Cancer. 2023 Sep 29;23:919. doi: 10.1186/s12885-023-11380-6 (PMC10540375; doi:10.1186/s12885-023-11380-6)
Supplement: Supplementary file 1 — Additional file 1: Online supplementary table 1. Institutional Review Board (IRB) and Institutional Ethics Committees (IEC). Online supplementary table 2. Patient disposition and treatment. Online supplementary table 3. Univariable analysis of associations between baseline patient demographic/clinicopathologic characteristics and long-term PFS. Online supplementary table 4. Univariable analysis of associations between baseline laboratory measurements and long-term PFS. Online supplementary table 5. Multivariable logistic regression model: associations between baseline characteristics and long-term PFS. [file 12885_2023_11380_MOESM1_ESM.docx]

**Online supplementary data**

**Identification of characteristics predictive of long-term survival with durvalumab or durvalumab plus tremelimumab in metastatic urothelial carcinoma**

Marie Alt^1,†^, Carlos Stecca^1^, Yian Lin^2^, Gbenga Kazeem^3^, Erik T. Goluboff^2^ and Srikala S. Sridhar^1,^*

^1^Princess Margaret Cancer Centre, Princess Margaret Hospital, University of Toronto, Toronto, Ontario, Canada

^2^AstraZeneca, Gaithersburg, Maryland, USA at the time of the analysis

^3^AstraZeneca, Cambridge, UK

^†^Current affiliation: Centre Hospitalier de Haguenau, Haguenau, France

## Online supplementary table 1 Institutional Review Board (IRB) and Institutional Ethics Committees (IEC)

## STUDY 10. List of IRBs/IECs

| **Site Number** | **Investigator Name** | **Name of IRB/IEC** |
| --- | --- | --- |
| 2000716 | Razak, Albiruni | University Health Network Research Ethics Board, Toronto, Ontario, Canada |
| 2000717 | Tsai, Frank | Western Institutional Review Board, Puyallup, WA, USA |
| 2000718 | Papadopoulos, Kyriakos | IntegReview Ethical Review Board, Austin, TX, USA |
| 2000719 | Hamid, Omid | Copernicus Group Independent Review Board, Research Triangle Park, NC, USA |
| 2000720 | Naing, Aung | MD Anderson Cancer Center Institutional Review Board, New York, NY, USA |
| 2000721 | Segal, Neil | Memorial Sloan Kettering Cancer Center Institutional Review Board, New York, NY, USA |
| 2000722 | Fishman, Mayer | Chesapeake IRB, Columbia, MD, USA |
| 2000915 | Chung, Ki | Greenville Health System Institutional Review Board, Greenville, SC, USA |
| 2000916 | Dowlati, Afshin | University Hospitals Case Medical Center Institutional Review Board, Cleveland, OH, USA |
| 2000945 | Smith, David | University of Michigan Medical School, Institutional Review Board, Ann Arbor, MI, USA |
| 2000964 | Luke, Jason | University of Chicago Institutional Review Board, Chicago, IL, USA |
| 2000969 | Leidner, Rom | Providence Health and Services Institutional Review Board, Portland, OR, USA |
| 2000998 | Plaxe, Steven | University of California San Diego Moores Cancer Center. La Jolla, CA, USA |
| 2001020 | Morris, John | University of Cincinnati Institutional Review Board - Medical Center, Cincinnati, OH, USA |
| 2001035 | Schneider, Reva | Mary Crowley Medical Research Center, Institutional Review Board, Dallas, TX, USA |
| 2001056 | Cho, Daniel | NYU School of Medicine Institutional Review Board, New York, NY, USA |
| 2001057 | Chow, Warren | Western Institutional Review Board, Puyallup, WA, USA |
| 2001122 | Villalobos, Victor | Western Institutional Review Board, Puyallup, WA, USA |
| 2001667 | Drescher, Charles | Western Institutional Review Board, Puyallup, WA, USA |
| 2002487* | Chung, Byung Ha | Gangnam Severance Hospital Institutional Review Board, Seoul, South Korea |
| 2002488 | Kang, Taek Won | Chonnam National University Hospital Institutional Review Board, Gwangju, South Korea |
| 2002489 | Kwon, Tae Gyun | Kyungpook National University Chilgok Hospital Institutional Review Board, Daegu, South Korea |
| 2002490 | Kim, Se Hyun | Seoul National University Bundang Hospital Institutional Review Board,  Seongnam-si, Gyeonggi-do, South Korea |
| 2002491 | Lee, Ji Youl | The Catholic University of Korea, Seoul St. Mary’s Hospital Institutional Review Board Seoul, South Korea |
| 2002492 | Shin, Sang Joon | Yonsei University Health System, Severance, Hospital Institutional Review Board, Seoul, South Korea |
| 2002493* | Keam, Bhumsuk | Seoul National University Hospital Institutional Review Board, Seoul, South Korea |
| 2002494 | Ha, Hong Koo | Pusan University Hospital Institutional Review Board, Busan, South Korea |
| 2002495 | Kim, Chun Il | Keimyung University Dongsan Medical Center Institutional Review Board, Daegu, South Korea |
| 2002496 | Park, Se Hoon | Samsung Medical Center Institutional Review Board, Seoul, South Korea |
| 2002497* | Lee, Jae Lyun | Asan Medical Center Institutional Review Board, Seoul, South Korea |
| 2002511* | Pieczonka, Christopher | Copernicus Group Independent Review Board, Research Triangle Park, NC, USA |
| 2002524* | Nikolinakos, Petros | Copernicus Group Independent Review Board, Research Triangle Park, NC, USA |
| 2002544* | Frank, Stephen | Ethics (Helsinki) Committee – Hadassah Medical Center, Kirvat Hadassah Jerusalem, Israel. Zip code: 9112001 |
| 2002547 | Kejzman, Daniel | Ethics (Helsinki) Committee – Meir Medical Center  59 Tchernichovsky Street, Kfar Saba, Israel. Zip code: 4428164 |
| 2002548* | Avivit Peer | Ethics (Helsinki) Committee – Rambam Health Care Campus  8 Ha'aliya Hashniya Street, Haifa, Israel. Zip code: 3109601 |
| 2002549* | Sarid, David | Ethics (Helsinki) Committee – Tel Aviv Sourasky Medical Center |
| 2002550 | Gabizon, Alberto | 6 Weizmann Street, Tel Aviv, Israel |
| 2002551* | Sella, Avishay | Ethics (Helsinki) Committee, Assaf Harofeh Medical Center  Zerifin, Be’er Ya’akov, Israel. Zip code: 7030000 |
| 2002561* | Bedke, Jens | Ethik-Kommission an der Medizinischen Fakultat der Eberhard-Karls-Universitat und am Universitatsklinikum Tubingen, Tubingen, Germany |
| 2002562* | Bogemann, Martin | Ethik-Kommission der Arztekammer Westfalen-Lippe und der Medizinischen Fakultat der Westfalischen Wilhelms-Universitat Munster  Munster, Germany |
| 2002563* | Grimm, Marc-Oliver | Ethik-Kommission der Friedrich-Schiller- Universitat Jena, Jena, Germany |
| 2002567 | Spira, Alexander | Copernicus Group Independent Review Board Research Triangle Park, NC, USA |
| 2002580 | Kalebasty, Arash Rezazadeh | Western Institutional Review Board, Puyallup, WA, USA |
| 2002581* | Park, Haeseong | Washington University School of Medicine Human Research Protection Office (HRPO), St. Louis, MO, USA |
| 2002583* | Tejwani, Sheela | Institutional Review Board – Henry Ford Health System, Detroit, MI, USA |
| 2002621 | Mahalingam, Devalingam | Northwestern University IRB, Chicago, IL, USA |
| 2002627 | Van Der Voet, Johannes | R&D – Institute of Learning, Research and Innovation, South Tees NHS Foundation Trust, James Cook University Hospital Middlesbrough, England, UK |
| 2002629 | Kristeleit, Rebecca | Joint Research Office (JRO), University College London Hospitals NHS Foundation Trust, London, England, UK |
| 2002630 | Powles, Thomas | Research Governance, Barts Health NHS Trust, Queen Mary University of London, London, England, UK |
| 2002631 | Syndikus, Isabella | Research and Innovation, Clatterbridge Cancer Centre NHS Foundation Trust, Wirral, England, UK |
| 2002632* | Jones, Robert | NHS Greater Glasgow & Clyde, Research & Development, West Glasgow Ambulatory Care Hospital, Glasgow, Scotland, UK |
| 2002636* | Arkenau, Hendrik-Tobias | HCA Healthcare R&D, Sarah Cannon Research Institute UK, London, England, UK |
| 2002641* | Protheroe, Andrew | Oxford University Hospitals NHS Foundation Trust, Oxford, England, UK |
| 2002645 | Mao, Shifeng | Copernicus Group Independent Review Board Research Triangle Park, NC, USA |
| 2002646 | Cetnar, Jeremy | OHSU Institutional Review Board, Portland, OR, USA |
| 2002647 | Lacombe, Louis | Jewish General Hospital Research Ethics Committee, Montreal, Quebec, Canada |
| 2002659 | Goel, Sanjay | BioMedical Research Alliance of New York, LLC/Institutional Review Board, Lake Success, NY, USA |
| 2002662* | Carthon, Bradley | Emory University Institutional Review Board Atlanta, GA, USA |
| 2002666 | Loriot, Yohann | Comite de Protection des Personnes - Ile de France III, Paris, France |
| 2002668 | Geoffrois, Lionel | Comite de Protection des Personnes - Ile de France III, Paris, France |
| 2002669 | You, Benoit | Comite de Protection des Personnes - Ile de France III, Paris, France |
| 2002670* | Vansteene, Damien | Comite de Protection des Personnes - Ile de France III, Paris, France |
| 2002671* | Gross-Goupil, Marine | Comite de Protection des Personnes - Ile de France III, Paris, France |
| 2002672 | Salas, Sebastien | Comite de Protection des Personnes - Ile de France III, Paris, France |
| 2002675* | Delva, Remy | Comite de Protection des Personnes - Ile de France III, Paris, France |
| 2002676 | Thiery-Vuillemin, Antoine | Comite de Protection des Personnes - Ile de France III, Paris, France |
| 2002681 | Cheng, Susanna | Research Ethics Office Human Research Protection Program, Sunnybrook Health Sciences Centre, Toronto, Ontario, Canada |
| 2002682* | Lerner, Rachel | Park Nicollet IRB, HealthPartners Institute St. Louis Park, MN, USA |
| 2002684 | Kim, Joseph | Yale University Institutional Review Board #2, 3, 4B, 5 – Human Investigation Committee I, II, III, IV, New Haven, CT, USA |
| 2002686* | Calvo, Emiliano | Hospital Madrid Sanchinarro, Madrid, Spain |
| 2002687 | Castellano, Daniel | Hospital Universitario, Madrid, Spain |
| 2002688 | Cervantes, Andres | Hospital **Clínico** Universitario de Valencia, Valencia, Spain |
| 2002689* | Duran, Ignacio | Hospital Virgen del Rocio, Sevilla, Spain |
| 2002690 | Gordoa, Theresa Alonso | Hospital Universitario Ramon y Cajal, Madrid, Spain |
| 2002691 | Mellado, Begona | Hospital Clinic i Provincial de Barcelona, Barcelona, Spain |
| 2002693 | Maroto, Jose Pablo | Hospital de la Santa Creu I Sant Pau, Barcelona, Spain |
| 2002695* | Garcia del Muro, Francisco | Institut Catala d’Oncologia, Barcelona, Spain |
| 2002697 | Milagro, Nuria Lainez | Complejo Hospitalario de Navarra, Pamplona, Navarra, Spain |
| 2002698 | Morales, Rafael | Hospital Vall Hebron, Barcelona, Spain |
| 2002702* | Friedlander, Terence | UCSF Institutional Review Board, San Francisco, CA, USA |
| 2002739 | Plimack, Elizabeth | Western Institutional Review Board, Puyallup, WA, USA |
| 2002741 | Ferrario, Cristiano | Jewish General Hospital Research Ethics Committee, Montreal, Quebec, Canada |
| 2002742 | Voortman, Johannes | Vrije Universiteit Medisch Centrum, Amsterdam, The Netherlands |
| 2002743 | van der Heijden, Michiel | Nederlands Kanker Instituut - Antoni van Leeuwenhoek Amsterdam (METC AVL), Amsterdam, The Netherlands. |
| 2002744* | Desar, Ingrid | CMO Regio Arnhem-Nijmegen, Nijmegen, The Netherlands |
| 2002745* | Coumou, Anna | METC ETZ TweeSteden, Tilburg, The Netherlands |
| 2002747* | Dasgupta, Anirudha | Copernicus Group Independent Review Board Research Triangle Park, NC, USA |
| 2002759 | Shum, Merrill | Copernicus Group Independent Review Board Research Triangle Park, NC, USA |
| 2002764* | Graham, Robert | Western Institutional Review Board, Puyallup, WA, USA |
| 2002886 | Drakaki, Alexandra | UCLA Office of the Human Research Protection Program (OHRPP), Los Angeles, CA, USA |
| 2002978* | Cobb, Patrick | Western Institutional Review Board, Puyallup, WA, USA |
| 2003001* | Pizzolato, Joseph | Mount Sinai Medical Center Institutional Review Board, Miami Beach, FL, USA |
| 2003016* | Vogelzang, Nicholas | Copernicus Group Independent Review Board Research Triangle Park, NC, USA |
| 2003039* | Winquist, Eric | Research Ethics Western Research, Western University, London, Ontario, Canada |
| 2003043* | Tosi, Diego | Comite de Protection des Personnes - Ile de France III, Paris, France |
| 2003044* | Flechon, Aude | Comite de Protection des Personnes - Ile de France III, Paris, France |
| 2003075* | Milowsky, Matthew | The University of North Carolina at Chapel Hill, Office of Human Research Ethics, Chapel Hill, NC, USA |
| 2003102* | Zakharia, Yousef | The University of Iowa, Human Subjects Office/Institutional Review Board (IRB), Iowa City, IA, USA |

* = only screen failures.

## Study 1108. List of IRBs/IECs

| **Site Number** | **Investigator Name** | **Name of IRB/IEC** |
| --- | --- | --- |
| 1002501 | Maio, Michele | Comitato Etico per la Sperimentazione Clinica Dei Medicinali dell'A.O.U. Senese di Siena |
| 1053601 | Marshall, John | Georgetown University Institutional Review Board |
| 1056201 | Antonia, Scott | Liberty Institutional Review Board |
| 1062001 | Lebbé, Céleste | Association pour la Recherche le Traitement et l'Enseignement en Oncologie a Saint- Louis (ARTEOS) |
| 1062001 | Lebbé, Céleste | Hôpital Saint Antoine, Comité de Protection des Personnes Ile de France V |
| 1093501 | Hwu, Wen-Jen | Texas Oncology Austin Brain Tumor Center |
| 1094301 | Khleif, Samir | Georgia Regents University |
| 1245501 | Lutzky, Jose | Mount Sinai Hospital Research Ethics Board |
| 1322701 | Brahmer, Julie | Johns Hopkins Institutional Review Board |
| 1351901 | Segal, Neil | Memorial Sloan Kettering |
| 1371101 | Curiel, Tyler | University of Texas Health Science Center Committee for the Protection of Human Subjects |
| 1371501 | Schöffski, Patrick | Universitaire Ziekenhuizen Leuven Gasthuisberg |
| 1371701 | Massard, Christophe | Hôpital Saint Antoine, Comité de Protection des Personnes Ile de France V |
| 1372001 | Ascierto, Paolo Antonio | Comitato Etico Istituto Nazionale per lo Studio e la Cura dei Tumori Fondazione G. Pascale |
| 2000042 | Hamid, Omid | Western Institutional Review Board |
| 2000043 | Gadgeel, Shirish | Western Institutional Review Board |
| 2000044 | Ou, Sai-Hong | University of California Davis Health System, Institutional Review Board |
| 2000044 | Ou, Sai-Hong | University of California, Irvine, Institutional Review Board |
| 2000045 | Butler, Marcus | University Health Network Research Ethics Board |
| 2000080 | Curigliano, Giuseppe | Comitato Etico Istituto Europeo di Oncologia e Centro Cardiologico Monzino |
| 2000089 | Sharma, Sunil | University of Utah Institutional Review Board |
| 2000090 | Zandberg, Dan | IRB-University of Maryland Medical Center |
| 2000108 | Gordon, Michael | Western Institutional Review Board |
| 2000112 | O'Donnell, Peter | University of Chicago Institutional Review Board |
| 2000113 | Sanborn, Rachel | Providence Health and Services Institutional Review Board |
| 2000124 | Ott, Patrick | Dana-Farber Cancer Institute Institutional Review Board |
| 2000126 | Eder, Joseph | Yale University School of Medicine, Human Investigation Committee |
| 2000133 | Wainberg, Zev | University of California at Los Angeles Office for the Protection of Research Subjects (OPRS) |
| 2000134 | Gutierrez, Martin | Western Institutional Review Board |
| 2000135 | Jamal, Rahima | Comité d'éthique de la recherche du CHUM |
| 2000136 | Nemunaitis, John | Mary Crowley Medical Research Center Institutional Review Board |
| 2000137 | Smith, David | University of Michigan Medical School Institutional Review Board |
| 2000166 | Sato, Takami | Thomas Jefferson University Institutional Review Board, Div. of Human Subjects Protection |
| 2000167 | Wakelee, Heather | Stanford University Institutional Review Board |
| 2000197 | Hoimes, Christopher | University Hospitals Case Medical Center, Institutional Review Board |
| 2000199 | Weiss, Jared | University of North Carolina, Office of Human Research Ethics, Biomedical Institutional Review Board |
| 2000199 | Weiss, Jared | Western Institutional Review Board |
| 2000206 | Jerusalem, Guy | CHU Sart Tilman-Comité d'Ethique Hospitalo-Facultaire Universitaire de Liège |
| 2000206 | Jerusalem, Guy | Universitair Ziekenhuis Leuven |
| 2000208 | Gianni, Luca | Comitato Etico dell'Ospedale San Raffaele |
| 2000209 | Keilholz, Ulrich | Landesamt für Gesundheit und Soziales Berlin, Geschäftsstelle der Ethik-Kommission des Landes Berlin |
| 2000210 | Jäger, Dirk | Ethikkommission der Universität Heidelberg |
| 2000211 | Cunningham, David | NRES Committee London - Surrey Borders |
| 2000213 | Middleton, Mark | NRES Committee London - Surrey Borders |
| 2000218 | Ciombor, Kristen | Western Institutional Review Board |
| 2000221 | Kim, Dong-Wan | Seoul National University Hospital Institutional Review Board |
| 2000228 | Kim, Sang-We | Asan Medical Center Institutional Review Board |
| 2000234 | Cunningham, David | NRES Committee London - Surrey Borders |
| 2000235 | Ahn, Myung-Ju | Samsung Medical Center Institutional Review Board |
| 2000410 | Goel, Sanjay | Biomedical Research Alliance of New York, Institutional Review Board |
| 2000437 | von Pawel, Joachim | Ethikkommission der Bayerischen Landesärztekammer |
| 2000438 | Romano, Gianpiero | Comitato Etico, Azienda Sanitaria Lecce |
| 2000439 | Rafii, Saeed | NRES Committee London - Surrey Borders |
| 2000675 | Shum, Merrill | Western Institutional Review Board |
| 2000677 | Mena, Raul | Providence Health and Services Institutional Review Board |
| 2000677 | Mena, Raul | Western Institutional Review Board |
| 2000678 | Powderly, II, John | Western Institutional Review Board |
| 2000679 | Shih, Kent | Western Institutional Review Board |
| 2000680 | Smith, Pamela | Western Institutional Review Board |
| 2000734 | Leach, Joseph | Quorum Review Institutional Review Board |
| 2000744 | Colon-Otero, Gerardo | Mayo Foundation Office for Human Research Protection |
| 2000747* | Bui, Lynne | Western Institutional Review Board |
| 2001023 | Spira, Alexander | Western Institutional Review Board |
| 2001024 | Blakely, Collin | Committee on Human Research |
| 2001061 | Nikolinakos, Petros | Western Institutional Review Board |
| 2001062 | Chung, Ki-Young | Greenville Health System. |
| 2001062 | Chung, Ki-Young | Greenville Hospital System University Medical Center |
| 2001077 | Lee, Jong-Seok | Seoul National University Bundang Hospital Institutional Review Board |
| 2001090 | Sadjadian, Parvis | Ethik-Kommission der Ärztekammer Westfalen-Lippe und der Medizinischen Fakultät der Westfälischen Wilhelms-Universität Münster |
| 2001109 | Zylla, Dylan | Park Nicollet Institute Institutional Review Board for HealthPartners Institute |
| 2001115 | Goldschmidt, Jerome | Western Institutional Review Board |
| 2001117 | Dasgupta, Anirudha | Western Institutional Review Board |
| 2001118 | Gold, Philip | Western Institutional Review Board |
| 2001185 | Goss, Glenwood | Ottawa Health Science Network Research Ethics Board |
| 2001190 | Braiteh, Fadi | Western Institutional Review Board |
| 2002156* | Lin, Chia-Chi | Research Ethics Committee, National Taiwan University Hospital |
| 2002165 | Su, Wen-Pin | Institutional Review Board National Cheng Kung University Hospital |
| 2002166* | Ha, Hong Koo | Pusan University Hospital Institutional Review Board |
| 2002177 | Grimm, Marc-Oliver | Ethik-Kommission der Friedrich-Schiller-Universität Jena |
| 2002181 | Kang, Taek Won | Chonnam National University Hospital Institutional Review Board |
| 2002222 | Goeminne, Jean-Charles | Comité d’Ethique - Clinique Sainte-Elisabeth |
| 2002222 | Goeminne, Jean-Charles | Universitair Ziekenhuis Leuven |
| 2002252 | Rottey, Sylvie | Commissie voor Medische Ethiek - UZ Gent |
| 2002346 | Powles, Thomas | London – Surrey Borders Research Ethics Committee |
| 2002371 | Mao, Shifeng | Western Institutional Review Board |

## Online supplementary table 2 Patient disposition and treatment.

|  | **OS ≥2 years** | **OS <2 years** |
| --- | --- | --- |
| Full analysis set (*N*) | 88 | 272 |
| Received treatment – *n* (%) | 88 (100) | 272 (100) |
| Completed treatment – *n* (%) | 62 (70.5) | 7 (2.6) |
| Discontinued treatment – *n* (%) | 26 (29.5) | 265 (97.4) |
| Disease progression | 15 (17.0) | 186 (68.4) |
| Adverse event | 8 (9.1) | 24 (8.8) |
| Death | 0 (0.0) | 33 (12.1) |
| Withdrawal by patient | 1 (1.1) | 10 (3.7) |
| Withdrawal of consent | 0 (0.0) | 9 (3.3) |
| Patient request | 0 (0.0) | 3 (1.1) |
| Investigator discretion | 1 (1.1) | 0 (0.0) |
| Other | 1 (1.1) | 0 (0.0) |

*OS* overall survival.

## Online supplementary table 3 Univariable analysis of associations between baseline patient demographic/clinicopathologic characteristics and long-term PFS.

| **Variable** | **PFS ≥2 years (*n* = 84)** | **PFS <2 years (*n* = 276)** | ***P* value^a^** |
| --- | --- | --- | --- |
| Sex – *n* (%) |  |  | 0.133 |
| Female | 16 (19.0) | 75 (27.2) |  |
| Male | 68 (81.0) | 201 (72.8) |  |
| Age, years – mean (SD) | 66.1 (8.8) | 65.4 (9.7) | 0.572 |
| Age group – *n* (%) |  |  | 0.404 |
| <50 y | 3 (3.6) | 17 (6.2) |  |
| ≥50 – <60 y | 14 (16.7) | 55 (19.9) |  |
| ≥60 – <70 y | 31 (36.9) | 113 (40.9) |  |
| ≥70 y | 36 (42.9) | 91 (33.0) |  |
| Race – *n* (%) |  |  | 0.542 |
| Asian | 12 (14.3) | 55 (19.9) |  |
| Black or African American | 1 (1.2) | 9 (3.3) |  |
| White | 60 (71.4) | 179 (64.9) |  |
| Other | 6 (7.1) | 19 (6.9) |  |
| Unknown | 5 (6.0) | 14 (5.1) |  |
| Smoking history – *n* (%) |  |  | 0.725 |
| Never | 33 (39.3) | 96 (34.8) |  |
| Former | 44 (52.4) | 151 (54.7) |  |
| Current | 7 (8.3) | 28 (10.1) |  |
| Unknown | 0 (0.0) | 1 (0.4) |  |
| Pack years smoked – mean (SD)^b^ | 25.2 (37.2) | 22.9 (29.6) | 0.949^c^ |
| ECOG PS – *n* (%) |  |  | <0.001 |
| 0 | 43 (51.2) | 84 (30.4) |  |
| 1 or 2 | 41 (48.8) | 192 (69.6) |  |
| Prior lines of treatment – *n* (%) |  |  | 0.022 |
| 0 | 4 (4.8) | 1 (0.4) |  |
| 1 | 55 (65.5) | 173 (62.7) |  |
| 2 | 21 (25.0) | 88 (31.9) |  |
| 3 | 4 (4.8) | 8 (2.9) |  |
| 4 | 0 (0.0) | 6 (2.2) |  |
| Prior surgery – *n* (%) |  |  | 0.022 |
| Yes | 72 (85.7) | 203 (73.6) |  |
| No | 12 (14.3) | 73 (26.4) |  |
| Prior radiotherapy – *n* (%) |  |  | 0.444 |
| Yes | 17 (20.2) | 67 (24.3) |  |
| No | 67 (79.8) | 209 (75.7) |  |
| Prior biologic, *n* (%) |  |  | 0.015 |
| Yes | 21 (25.0) | 38 (13.8) |  |
| No | 63 (75.0) | 238 (86.2) |  |
| Prior immunotherapy, *n* (%) |  |  | 0.125 |
| Yes | 0 (0.0) | 10 (3.6) |  |
| No | 84 (100.0) | 266 (96.4) |  |
| Prior cisplatin-based regimen, *n* (%) |  |  | 0.301 |
| Yes | 60 (71.4) | 202 (73.2) |  |
| No | 17 (20.2) | 41 (14.9) |  |
| Missing | 7 (8.3) | 33 (12.0) |  |
| Prior carboplatin-based regimen, *n* (%) |  |  | 0.376 |
| Yes | 28 (33.3) | 79 (28.6) |  |
| No | 29 (34.5) | 107 (38.8) |  |
| Missing | 27 (32.1) | 90 (32.6) |  |
| PD-L1 status, *n* (%)^d^ |  |  | 0.001 |
| Low or negative | 25 (29.8) | 141 (51.1) |  |
| High | 51 (60.7) | 116 (42.0) |  |
| Missing | 8 (9.5) | 19 (6.9) |  |
| Time from initial diagnosis to study entry, months – mean (SD) | 35.1 (41.3) | 27.1 (27.1) | 0.055^c^ |
| Lymph node-only involvement, *n* (%) |  |  | <0.001 |
| Yes | 22 (26.2) | 22 (8.0) |  |
| No | 62 (73.8) | 254 (92.0) |  |
| Visceral disease, *n* (%) |  |  | <0.001 |
| Yes | 42 (50.0) | 219 (79.3) |  |
| No | 42 (50.0) | 57 (20.7) |  |

^a^*P* value was estimated from t-test for continuous variables, chi-squared for categorical variables where the number of patients is at least 5 in each comparison group, and Fisher’s exact test for categorical variables where the number of patients is less than 5 in any comparison group. Comparisons were based on non-missing data.

^b^Number of packs per day × no. of years smoked. *n* = 72 for the OS ≥2 years subgroup and *n* = 199 for the OS <2 years subgroup.

^c^*P*-value was obtained from t-test based on log-transformed data.

^d^PD-L1 expression was assessed by immunohistochemistry using the VENTANA PD-L1 (SP263) Assay. PD-L1 expression was defined as “high” if ≥25% of tumour cells or ≥25% of tumour-infiltrating immune cells had PD-L1 membrane staining. PD-L1 expression was defined as “low or negative” if <25% of both tumour cells and immune cells had membrane staining for PD-L1.

*ECOG PS* Eastern Cooperative Oncology Group performance status, *PD-L1* programmed cell death ligand-1, *PFS* progression-free survival, *SD* standard deviation.

## Online supplementary table 4 Univariable analysis of associations between baseline laboratory measurements and long-term PFS.

| **Variable^a^** | **PFS ≥2 years (*n* = 84)** | **PFS <2 years (*n* = 276)** | ***P* value** |
| --- | --- | --- | --- |
| Haemoglobin level (g/dL) | 12.1 (1.8) | 11.2 (1.4) | <0.001 |
| Absolute neutrophil count (10^3^/μL) | 4.7 (2.5) | 6.5 (4.4) | <0.001^b^ |
| Absolute monocyte count (10^3^/μL) | 0.6 (0.3) | 0.7 (0.4) | 0.019^b^ |
| Absolute lymphocyte count (10^3^/μL) | 1.3 (0.6) | 1.3 (0.6) | 0.357^b^ |
| Absolute eosinophil count (10^3^/μL) | 0.2 (0.2) | 0.2 (0.4) | 0.077^b^ |
| Neutrophil-lymphocyte ratio | 4.3 (3.9) | 6.5 (6.9) | <0.001^b^ |
| Neutrophils/leukocytes | 66.5 (9.5) | 70.9 (10.6) | 0.002^b^ |
| Creatinine clearance (mL/min) | 68.6 (24.9) | 69.0 (24.9) | 0.897 |
| Lactate dehydrogenase level (U/L) | 258.7 (125.2) | 366.9 (517.7) | 0.010^b^ |

^a^Values for laboratory measurements are mean (standard deviation).

^b^P value was obtained from t-test based on log-transformed data.

PFS progression-free survival.

## Online supplementary table 5 Multivariable logistic regression model: associations between baseline characteristics and long-term PFS.

| **Variable^a^** | **Odds ratio (95% CI)** | **P value** |
| --- | --- | --- |
| ECOG PS |  |  |
| 0 | (Reference) |  |
| 1 or 2 | 0.60 (0.33–1.09) | 0.094 |
| Prior surgery |  |  |
| No | (Reference) |  |
| Yes | 1.71 (0.80–3.65) | 0.167 |
| Prior biologic |  |  |
| No | (Reference) |  |
| Yes | 1.92 (0.94–3.91) | 0.074 |
| PD-L1 status |  |  |
| Low or negative | (Reference) |  |
| High | 1.90 (1.05–3.41) | 0.032 |
| Lymph node-only involvement |  |  |
| No | (Reference) |  |
| Yes | 1.30 (0.49–3.47) | 0.595 |
| Visceral disease |  |  |
| No | (Reference) |  |
| Yes | 0.57 (0.26–1.23) | 0.151 |
| Haemoglobin level | 1.18 (0.96–1.46) | 0.123 |
| Absolute neutrophil count^a^ | 0.27 (0.08–0.99) | 0.048 |
| Absolute monocyte count^a^ | 1.79 (0.66–4.81) | 0.250 |
| Neutrophil-lymphocyte ratio^a^ | 0.61 (0.17–2.18) | 0.448 |
| Neutrophils/leukocytes^a^ | 62.4 (0.29–13000) | 0.131 |
| Lactate dehydrogenase level^a^ | 0.82 (0.46–1.48) | 0.514 |

^a^Data were log-transformed for the logistic regression analysis. Prior lines of treatment were not included in the model due to small numbers of patients who had received 0, 3, or 4 prior lines of treatment.

ECOG PS Eastern Cooperative Oncology Group performance status, PD-L1 programmed cell death ligand-1, PFS progression-free survival.
